# Different computed tomography patterns of Coronavirus Disease 2019 (COVID-19) between survivors and non-survivors

**DOI:** 10.1038/s41598-020-68057-4

**Published:** 2020-07-09

**Authors:** Feng Pan, Chuansheng Zheng, Tianhe Ye, Lingli Li, Dehan Liu, Lin Li, Richard L. Hesketh, Lian Yang

**Affiliations:** 1 Department of Radiology, Union Hospital, Tongji Medical College, Huazhong University of Science and Technology, Jiefang Avenue 1277#, Wuhan, 430022 China; 2 Hubei Province Key Laboratory of Molecular Imaging, Wuhan, 430022 China; 3 Department of Radiology, University College London Hospital, 235, Euston Road, London, NW1 2BU UK

**Keywords:** Viral infection, Computed tomography

## Abstract

This study aimed to compare the chest computed tomography (CT) findings between survivors and non-survivors with Coronavirus Disease 2019 (COVID-19). Between 12 January 2020 and 20 February 2020, the records of 124 consecutive patients diagnosed with COVID-19 were retrospectively reviewed and divided into survivor (83/124) and non-survivor (41/124) groups. Chest CT findings were qualitatively compared on admission and serial chest CT scans were semi-quantitively evaluated between two groups using curve estimations. On admission, significantly more bilateral (97.6% vs. 73.5%, p = 0.001) and diffuse lesions (39.0% vs. 8.4%, p < 0.001) with higher total CT score (median 10 vs. 4, p < 0.001) were observed in non-survivor group compared with survivor group. Besides, crazy-paving pattern was more predominant in non-survivor group than survivor group (39.0% vs. 12.0%, p < 0.001). From the prediction of curve estimation, in survivor group total CT score increased in the first 20 days reaching a peak of 6 points and then gradually decreased for more than other 40 days (R^2^ = 0.545, p < 0.001). In non-survivor group, total CT score rapidly increased over 10 points in the first 10 days and gradually increased afterwards until ARDS occurred with following death events (R^2^ = 0.711, p < 0.001). In conclusion, persistent progression with predominant crazy-paving pattern was the major manifestation of COVID-19 in non-survivors. Understanding this CT feature could help the clinical physician to predict the prognosis of the patients.

## Introduction

Since December 2019, an outbreak of coronavirus disease 2019 (COVID-19) has emerged in Wuhan, China^1,2^. Subsequently, the disease has spread worldwide with a total infected population of more than 6.5 million reported on 5th June 2020^3^. The pathogen was confirmed as a novel beta-coronavirus, which has demonstrated rapid human-to-human transmission with a median incubation period of 3 days^4,5^. Recent data also suggest a higher transmission capability of this virus than the previously reported coronaviruses^3,6^.

The clinical characteristics and laboratory findings of COVID-19 patients have been reported including non-specific fever and cough symptoms and lymphopenia^2,4,7–9^. Real-time reverse transcription-polymerase chain reaction (RT-PCR) test has a relatively high false-negative rate (29%) for COVID-2019 diagnosis, so chest computed tomography (CT) is recommended as the major screen modality with a higher sensitivity of 97% and faster performance^10–13^. In Hubei province, the centre of the outbreak in China, the clinical diagnostic criteria were only dependent on chest CT scan, instead of the RT-PCR test before 19 February 2020^14^. However, the value of the consecutive CT scans for monitoring disease progression was still unclear.

Previous studies suggested a typical time course of CT findings in survivors with COVID-19, in which initial progression was followed by recovery, the latter starting after about 2 weeks^15–17^. Case series have associated severe and critical COVID-19 with more diffuse lung involvement, development of acute respiratory distress syndrome (ARDS), and multi-organ failure^7,18–20^. Using a case–control design, this study aims to identify the differentiating CT features and compare the temporal evolution of pulmonary involvement between recovered and died patients with COVID-19.

## Materials and methods

### Patients and groups

**Figure 1:**
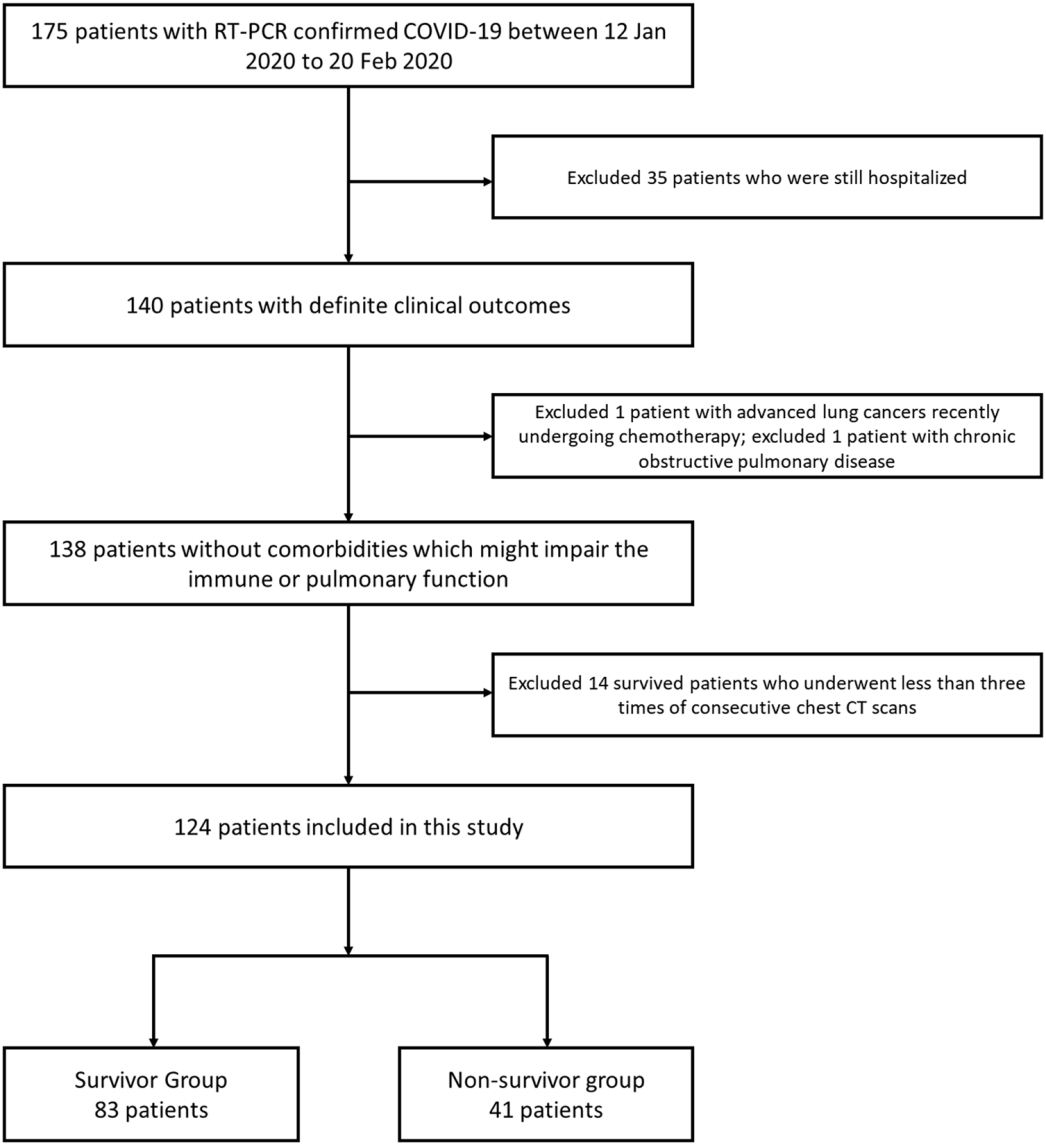
Flowchart of inclusion of the patients.

175 consecutive records of hospitalized patients with RT-PCR confirmed COVID-19 were reviewed retrospectively for the period from 12 January 2020 to 20 February 2020 in this single-centre (Union Hospital, Wuhan, China). The inclusion criteria included: (1) with definite clinical outcomes (discharge or death events); (2) no comorbidities which might impair the immune or pulmonary function, such as recent chemotherapy and chronic obstructive pulmonary disease; (3) with more than three times of chest CT scans in the course for sufficient estimation of radiological patterns, unless fatal ARDS occurred resulting in impossibility to carry out the consecutive chest CT scans. Eventually, 124 patients were included and divided into two groups: survivor group (discharged patients, n = 83, including 21 patients who were preliminarily reported in the previous study^15^) and non-survivor group (died patients, n = 41) (Fig. 1). Clinical data (e.g. initial symptoms, past medical history, etc.) and serial chest-CT data in the follow-up (extended until 30 March 2020 in survivor group) were retrieved through the institutional electronic patient database. Diagnostic, isolation, grades of the disease severities (non-ARDS and ARDS), treatment, and discharge criteria were based on the published standard protocols from the continuously-updated National Health Commission of the People’s Republic of China^14^.

### Chest CT scan protocols

Chest CT scans were performed using two commercial multi-detector CT scanners (Philips Ingenuity Core128, Philips Medical Systems, Best, Netherlands; SOMATOM Definition AS, Siemens Healthineers, Erlangen, Germany) during a single breath-hold. The low-dose mode was set up with a tube voltage of 120 kVp and automatic tube current modulation. From the raw data, CT images were reconstructed as 1.5 mm thick axial slices and increment of 1.5 mm in transverse slice orientation with either hybrid iterative reconstruction (iDose level 5, Philips Healthcare, Best, The Netherlands) or a pulmonary B70F kernel (Siemens Healthineers, Erlangen, Germany).

### Chest CT estimation

The abnormal radiological findings of CT reported using internationally standard nomenclature^21–23^. CT abnormalities included ground-glass opacity (GGO), crazy-paving pattern, and consolidation. The distribution of abnormalities was also noted as being predominantly subpleural (involving mainly the subpleural one-third of the lung), random (without predilection for subpleural or central regions), or diffuse (continuous involvement without respect to lung segments)^24^. A conventional semi-quantitative scoring system was used to evaluate the pulmonary involvement area of all these abnormalities^15,25^. There was a score of 0–5 for each lobe on the following: 0—no involvement; 1, < 5% involvement; 2, 6–25% involvement; 3, 26–49% involvement; 4, 50–75% involvement; 5, > 75% involvement. The total CT score was the sum of the score of each lobe and ranged from 0 (no involvement) to 25 (maximum involvement). The analysis was performed using the institutional digital database system (Vue PACS, version 11.3.5.8902, Carestream Health, Oakville, Canada) by two radiologists (CZ and LY, who had 26 and 22 years of experience in thoracic radiology, respectively) and the decisions were reached in consensus. All radiologists were blinded to the groups and clinical progress of the patients to avoid information bias.

### Statistical analysis

Statistical analysis was performed using IBM SPSS Statistics Software (version 24; IBM, New York, USA). Quantitative data were presented as median with inter-quartile range (IQR) and qualitative data were presented as the percentage of the total unless otherwise specified. The comparisons of the quantitative data were statistically evaluated using the Mann–Whitney U test, according to the non-normal distribution assessed by the Shapiro–Wilk test. The comparisons of qualitative data were evaluated using the Chi-square test or Fisher’s exact test. The dynamic total CT score with time from symptom onset was quantitatively assessed by using the SPSS curve estimation module^15^. A p value of < 0.05 was defined as having statistical significance.

## Results

### Basic characteristics

The median age of the patients was 56 years (IQR 38–68 years) with an approximately equal male to female ratio (63:61). The median age of patients was significantly higher in non-survivor compared to non-survivors (69 years vs. 43 years, p < 0.001). The percentage of males was 38.6% and 75.6% in survivor and non-survivor groups, respectively (p < 0.001) (Table 1). Non-survivors were also more likely to have a history of hypertension, diabetes, and coronary heart disease than survivors (p < 0.05) (Table 1). Fever and cough were the most common initial symptoms (85.5% and 65.3%, respectively). Chest distress was significantly more inclined to occur in non-survivors (p < 0.001) (Table 1). There was no significant difference in the period of admission from symptom onset between survivor and non-survivor groups (8 days vs. 9 days, p = 0.422) (Table 1). The median survival period of non-survivor group after admission was 14 days (IQR 8–22 days) from admission, while the median hospitalized period in survivor group was 18 days (IQR 12–27 days) (p = 0.068). The survivors underwent more times of chest CT scans than non-survivors (4 vs. 2, p < 0.001) with a significantly longer duration (6 days vs. 5 days, p = 0.001) (Table 1). All non-survivors aggravated to ARDS after a median of 11 days (8–14 days) from symptom onset, while only one patient aggravated to ARDS in survivor group.

**Table 1:** Basic characteristics and clinical outcomes.

	Total, n = 124	Survivor group, n = 83	Non-survivor group, n = 41	p value
Age (years) (IQR)	56 (38–68)	43 (34–61)	69 (63–78)	**< 0.001**
**Sex**				
Male	63 (50.8)	32 (38.6)	31 (75.6)	**< 0.001**
Female	61 (49.2)	51 (61.4)	10 (24.4)	
**Medical history**				
Hypertension	18 (14.5)	2 (2.4)	16 (39.0)	**< 0.001**
Diabetes	5 (4.0)	1 (1.2)	4 (9.8)	**0.041**
Coronary heart disease	8 (6.5)	1 (1.2)	7 (17.1)	**0.002**
**The initial symptoms of onset**				
Fever	106 (85.5)	73 (88.0)	33 (80.5)	0.267
Low grade fever (37.5–38.0 °C)	29 (23.4)	23 (27.7)	6 (14.6)	0.103
Moderate fever (38.1–39.0 °C)	47 (37.9)	34 (41.0)	13 (31.7)	
High grade fever (> 39.1 °C)	30 (24.2)	16 (19.3)	14 (34.1)	
Cough	81 (65.3)	54 (65.1)	27 (65.9)	0.930
Expectoration	43 (34.7)	26 (31.3)	17 (41.5)	0.264
Diarrhea	17 (13.7)	9 (10.8)	8 (19.5)	0.187
Chest distress	16 (12.9)	4 (4.8)	12 (29.3)	**< 0.001**
Myalgia	13 (10.5)	6 (7.2)	7 (17.1)	0.092
**Severity grades**				
Non-ARDS	82 (66.1)	82 (98.8)	0 (0.0)	**< 0.001**
ARDS	42 (33.9)	1 (1.2)	41 (100.0)	
Time of admission from symptom onset (days) (IQR)	8 (5–11)	8 (5–11)	9 (5–13)	0.422
Hospitalized period (days) (IQR)	17 (11–24)	18 (12–27)	14 (8–22)	0.068
Period of CT follow-up from symptom onset (days) (IQR)	32(20–46)	39 (27–52)	21(12–28)	0.118
Numbers of adjacent chest CT scans (days) (IQR)	4 (2–5)	4 (4–5)	2 (1–2)	**< 0.001**
Interval between adjacent chest CT scans (days) (IQR)	6 (5–12)	6 (5–13)	5 (3–9)	**0.001**

Multiple biochemical and haematological parameters differed significantly between the two groups such as lymphocyte count, neutrophil count, and C-reactive protein (CRP) (p < 0.05) (Table 2).

**Table 2:** Initial laboratory investigations on admission.

	Normal reference range	Total, n = 124	Survivor group, n = 83	Non-survivor group, n = 41	p value
White blood cell (× 10^9^/L) (IQR)	3.50–9.50	5.05 (3.91–7.04)	4.84 (3.78–5.77)	6.81 (4.79–10.91)	**< 0.001**
Neutrophil (× 10^9^/L) (IQR)	1.80–6.30	3.37 (2.47–6.13)	2.96 (2.24–3.97)	6.45 (3.83–9.70)	**< 0.001**
Lymphocyte (× 10^9^/L) (IQR)	1.10–3.20	0.94 (0.73–1.39)	1.17 (0.84–1.55)	0.73 (0.51–1.01)	**< 0.001**
Lymphocyte percentage (%) (IQR)	20.0–50.0	19.8 (10.7–30.1)	26.2 (18.2–33.2)	9.5 (5.9–17.8)	**< 0.001**
Hemoglobin (g/L) (IQR)	115–150	131 (121–143)	129 (121–142)	136 (121–144)	0.276
Platelet (× 10^9^/L) (IQR)	125–350	164 (130–207)	174 (139–216)	153 (125–186)	0.074
C-reactive protein (mg/L) (IQR)	0.00–8.00	16.60 (7.50–76.23)	10.85 (5.76–24.80)	78.11 (53.54–110.78)	**< 0.001**
Total bilirubin (μmol/L) (IQR)	3.0–20	10.2 (8.5–14.6)	9.6 (8.3–12.5)	11.9 (9.2–20.9)	**0.023**
Alanine aminotransferase (U/L) (IQR)	5–35	31 (21–50)	28 (17–47)	33 (23–56)	0.196
Aspartate aminotransferase (U/L) (IQR)	8–40	33 (24–50)	27 (22–38)	48 (36–64)	**< 0.001**
Lactate dehydrogenase (U/L) (IQR)	109–245	330 (202–520)	222 (181–338)	490 (363–636)	**< 0.001**
Albumin (g/L) (IQR)	33.0–55.0	33.9 (28.7–38.5)	36.1 (33.6–39.7)	28.1 (26.1–31.5)	**< 0.001**
Serum creatinine (μmol/L) (IQR)	41.0–81.0	72.0 (57.6–89.2)	68.0 (55.3–81.4)	78.3 (61.3–109.8)	**0.005**
d-dimer (mg/L) (IQR)	0.00–0.50	0.56 (0.26–1.98)	0.30 (0.22–0.53)	1.98 (0.75–8.00)	**< 0.001**

### Comparison of major CT findings between two groups

All 124 patients underwent a total of 436 chest CT scans with a median interval between adjacent scans of 6 days (IQR 5–12 days) (Table 1). 363 CT scans (363/436, 83.3%) were performed in 83 survivors, while 73 CT scans (73/436, 16.7%) were performed in the 41 non-survivors.

On admission, bilateral lung involvement was more common in non-survivors than survivors (97.6% vs. 73.5%, p = 0.001) (Table 3). Subpleural distribution was more inclined to be observed in survivors compared with non-survivor group (69.9% vs. 43.9%, p = 0.005), while diffuse distribution was more common in non-survivor group compared with survivor group (39.0% vs. 8.4%) (p < 0.001) (Table 3). GGO (83.1%), consolidation (60.5%), and crazy-paving pattern (42.7%) were the major CT findings in both groups, while the crazy-paving pattern was more common in non-survivor group than survivor group (65.9% vs. 31.3%, p < 0.001) (Table 3). On admission, consolidation predominated in survivor group (37.3%), but crazy-paving pattern predominated in non-survivor group compared with survivor group (39.0% vs. 12.0%, p = 0.001) (Table 3). Besides, the total CT score was significantly higher in non-survivor group than survivor group (a median of 10 vs 4, p < 0.001).

**Table 3:** Major CT findings on admission.

	Total, n = 124	Survivor group, n = 83	Non-survivor group, n = 41	p value
**Pulmonary involvement**				
No involvement	3 (2.4)	3 (3.6)	0 (0.0)	0.550
Unilateral	20 (16.1)	19 (22.9)	1 (2.4)	**0.003**
Bilateral	101 (81.5)	61 (73.5)	40 (97.6)	**0.001**
**Distribution of pulmonary lesions**				
No lesion	3 (2.4)	3 (3.6)	0 (0.0)	0.550
Subpleural	76 (61.3)	58 (69.9)	18 (43.9)	**0.005**
Random	22 (17.7)	15 (18.1)	7 (17.1)	0.891
Diffuse	23 (18.5)	7 (8.4)	16 (39.0)	**< 0.001**
**Major CT findings**				
GGO	103 (83.1)	69 (83.1)	34 (82.9)	0.977
Consolidation	75 (60.5)	48 (57.8)	27 (65.9)	0.390
Crazy-paving pattern	53 (42.7)	26 (31.3)	27 (65.9)	**< 0.001**
**Predominant CT findings**				
No lesions	3 (2.4)	3 (3.6)	0 (0.0)	0.550
GGO	27 (21.8)	23 (27.7)	4 (9.8)	**0.023**
Crazy-paving pattern	26 (21.0)	10 (12.0)	16 (39.0)	**0.001**
Consolidation	44 (35.5)	31 (37.3)	13 (31.7)	0.537
Mixed	24 (19.4)	16 (19.3)	8 (19.5)	0.975
Total CT score (IQR)	5 (2–10)	4 (2–7)	10 (5–13)	**< 0.001**

### Dynamic estimation of pulmonary involvement between two groups

**Figure 2:**
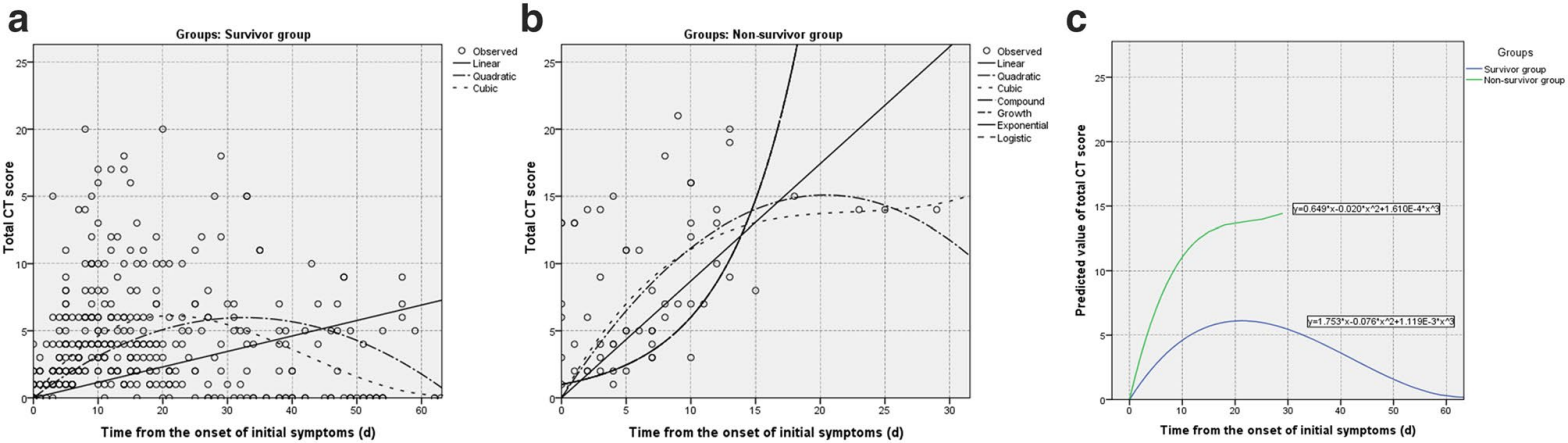
Curve estimations between survivor and non-survivor groups. (**a**) The curve estimations involved linear, quadratic, and cubic fitting, in which cubic fitting demonstrated the optimal equation (R^2^ = 0.545, p < 0.001); (**b**) the curve estimations involved linear, quadratic, cubic, compound, growth, exponential, and logistic fitting, in which cubic fitting demonstrated the optimal equation (R^2^ = 0.711, p < 0.001); (**c**) The comparison of optimal fitting curves between survivor and non-survivor groups (Equations of $\mathrm{y}=1.753\times \mathrm{x}-0.076\times \mathrm{x}^2+1.119\mathrm{E}-3\times \mathrm{x}^3$ and $\mathrm{y}=0.649\times \mathrm{x}-0.020\times \mathrm{x}^2+1.610\mathrm{E}-4\times \mathrm{x}^3$, respectively). All images were obtained from SPSS 24.0 software.

**Figure 3:**
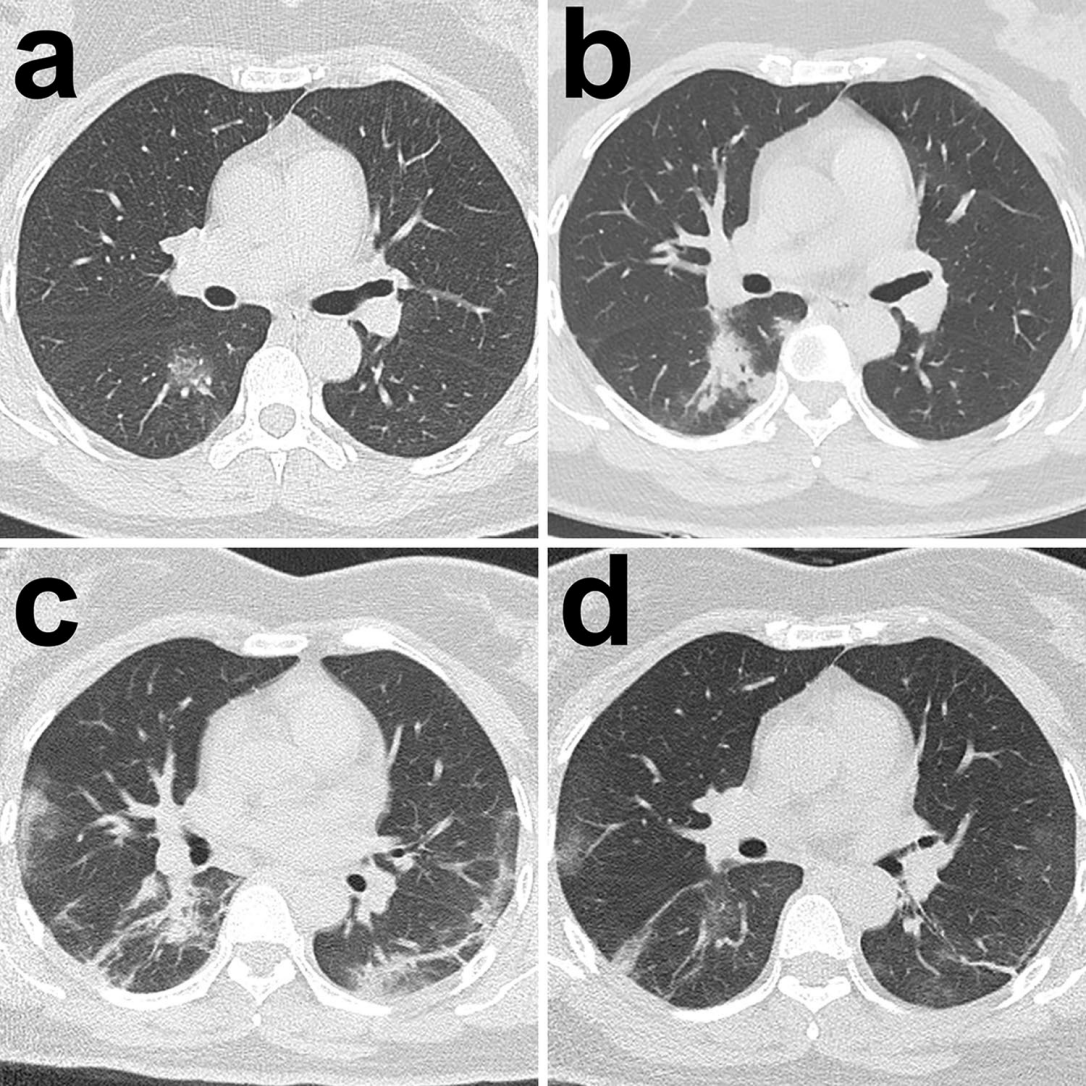
Typical radiological evolution of survivors. Images from a patient presenting with sudden fever (38.8 °C) for four days. (**a**) At presentation (day 4), a small region of GGO was demonstrated in the right lower lobe on CT scan and the RT-PCR test was performed afterwards; (**b**) on admission after confirming COVID-19 (day 9), the previous GGO became more consolidated with more surrounding subpleural lesions; (**c**) day 13, more bilateral subpleural GGO and consolidation were observed, and the previous consolidation was partially absorbed; (**d**) day 18, most of the lesions were absorbed while only some residual GGO and parenchymal bands could be observed. All images have the same window level of − 600 and window width of 1,600.

**Figure 4:**
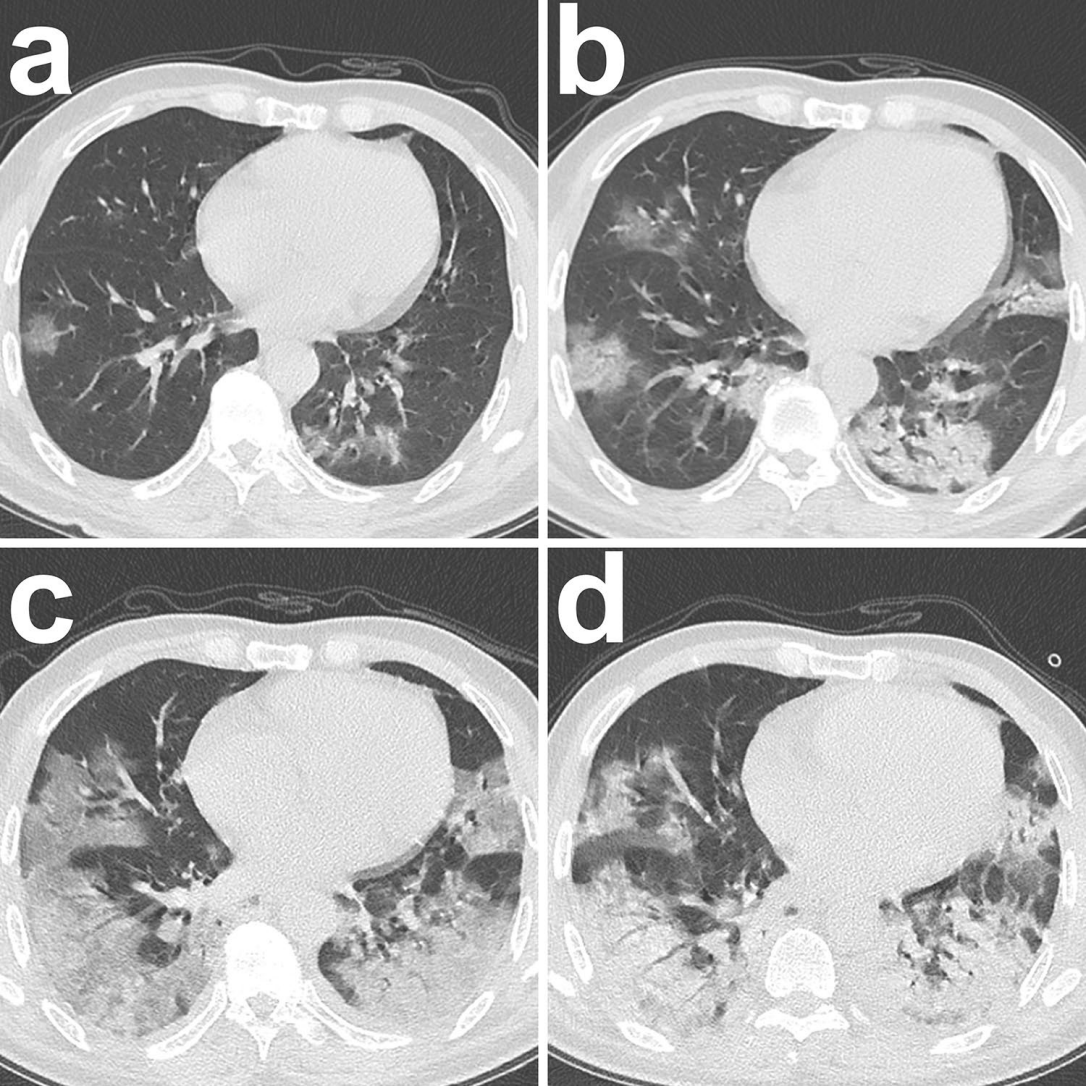
Typical radiological evolution of non-survivors. Images from a patient presenting with 3 days of sudden fever (38.0 °C). (**a**) At presentation (day 3), subpleurally distributed GGO with crazy-paving pattern was demonstrated in the bilateral lungs and the RT-PCR test was performed afterwards; (**b**) on admission after confirming COVID-19 (day 7), the previous GGO became more enlarged with the crazy-paving pattern and partially consolidation could be observed. Afterwards, progressive respiratory distress occurred. (**c**) day 12, diffuse bilateral lung involvement with extensive GGO and partial consolidation was observed. (**d**) Day 18, a similar area of pulmonary involvement with predominant consolidation was observed. The patient eventually died on day 22 due to refractory respiratory failure. All images have the same window level of − 600 and window width of 1,600.

Based on the analysis, the cubic model demonstrated the best fitting in both the survivor and non-survivor groups (R^2^ = 0.545 and 0.711, respectively; p < 0.001, each) (Fig. 2a,b; SI Table S1 online). The optimal fitting equations were demonstrated in Fig. 2c. From the optimal fitting, in survivor group the total CT score gradually increased in the first 20 days with a peak value of 6 and then gradually decreased afterwards lasting for more than another 40 days (Fig. 2c). The typical CT manifestation was changed from subpleural GGO to enlarged consolidation with time which was gradually absorbed afterwards leaving residual GGO and parenchymal bands (Fig. 3). But in non-survivor group, the total CT score rapidly increased in the first 10 days and eventually approached 15 until ARDS occurred (Fig. 2c). From the dynamic CT images, the persistently progressive pulmonary lesions from GGO with crazy-paving pattern to bilaterally extensive consolidation could be observed (Fig. 4).

## Discussion

This study compared the temporal changes in CT manifestations between survivors and non-survivors with COVID-19. It demonstrated the pulmonary involvement of subpleural GGO and sequential consolidation gradually progressed reaching the peak after 20 days since symptom onset in survivors. Afterwards, the lesions started to be absorbed lasting for more than 40 days. In contrast, non-survivors demonstrated more rapid and persistent progression with more extensive bilateral lesions until ARDS occurred. Crazy-paving pattern was more predominant in non-survivors on admission compared with survivors.

In accordance with the previous studies, old patients (69 years, IQR 63–78 years) with more comorbidities such as hypertension, diabetes, and coronary heart disease were more inclined to develop fatal ARDS^4,8^. Initial symptoms were similar between survivor and non-survivor groups, whilst chest distress was more common in non-survivor group. Patients in non-survivor group underwent a progressive phase which culminated in the development of ARDS after a median period of three weeks from symptom onset. As a case–controlled study, the mortality rate of ARDS caused by COVID-19 could not be evaluated, but from a previous study, it was reported mortality of 61.5%^26^.

Initial laboratory investigations on admission showed multiple haematological and biochemical abnormalities which were significantly different between survivor and non-survivor groups. This can be attributed to the systematic inflammation reaction and pulmonary vascular endothelial damage caused by a severe viral infection, similar to the systemic response seen in other types of severe pneumonia^8,24–28^. It has been postulated that COVID-19 could also damage T lymphocytes, thus, significant lymphopenia was probably a risk factor leading to the deterioration of patients' immune function and more rapid disease progression^7,8,26,29^. In addition, the increased levels of CRP, lactate dehydrogenase, and d-dimer could also be indicators for development of ARDS, as reported in other types of pneumonia^23,25,27,30^.

In the early stage of COVID-19, subpleural GGO was the predominant finding^15–17,20,31^. But in this study, patients were hospitalized after a median period of 8 and 9 days after the onset of symptoms in survivor and non-survivor groups, respectively, at which time the predominant findings in both groups corresponded with the progressive stage^15^. Thus, GGO was not the predominant finding in both groups but the consolidation and crazy-paving pattern. Compared with the survivors, it demonstrated the predominant CT demonstration of crazy-paving pattern in non-survivor group on admission was a major difference except for more diffuse and bilateral distributions. Pathologically, GGO may be an indicator of alveolar oedema and proteinaceous exudates^32^. As the disease progresses, increasing alveolar oedema, exudates, and lymphocyte infiltrates fill the interstitial space leading to the radiological demonstration of diffuse “crazy-paving pattern”^22,28,33,34^. Subsequent ARDS and potentially fatal respiratory failure developed as a result of diffuse alveolar oedema with loss of alveolar epithelium^22,34^. Thus, it was speculated large area of crazy-paving pattern was probably a CT indicator of poor prognosis.

Considering the heterogeneities of the scan time among the patients, longitudinal comparisons were not appropriate. Thus, the curve estimation was used to statistically compare the temporal evolution of the disease between two groups. Being different from the static comparison of chest CT on admission using the logistic module, curve estimation could analyze the dynamic patterns of the pulmonary involvement with time^19,35^. Thus, it could provide a more composite comprehension of the time course in COVID-19 between survivors and non-survivors. As a result, it demonstrated a gradual resolution of abnormalities after a maximal total CT score of 6 at 20 days, longer than 10 days reported in the previous report^15^. It might be ascribed to a limited sample size in the previous study, which probably underestimated the recovery duration of COVID-19. Compared with survivor groups, the total CT score in non-survivor group demonstrated a more rapid increase in the first 10 days with a higher value of more than 10 points. Although the previous study showed the feasibility of making CT score as an indicator of prognosis, it did not demonstrate the dynamic changes of CT score in the whole course^19^. This study revealed the total score persistently elevated to a higher level close to 15 points without any decrease in non-survivor group, until the ARDS occurred with the following death events. From one pathological study in severe acute respiratory syndrome (SARS), it found the long duration of illness resulted from the severe fibrosis and organization^28^. Considering the partial homology of SARS and COVID-19, it might explain why the lesions were rarely absorbed in non-survivors with COVID-19. This is another major difference between the two groups in the course, associated with the refractory feature of the critical COVID-19 under the present treatment protocols^14^.

This study has limitations. Firstly, as a retrospective study, chest CT was used by the physician based on the clinical necessity and the status of the patient, so the heterogeneities of scanning time made it impossible to perform a conventional longitudinal comparison between two groups with regular intervals. Second, CT was not clinically feasible for patients after developing ARDS so not enough CT information was provided in the course of ARDS. Consequently, the majority of CT scans were performed in mild disease (363/436, 83.3%). To avoid data heterogeneity, the comparison of chest CT between two groups was only performed on admission due to a similar period from symptom onset and the curve estimation was used to evaluate the comprehensive trend of pulmonary involvement between two groups. Third, the multi-variate regression or propensity matching involving the CT, clinical, and laboratory parameters was not performed owing to the limited sample size and a relatively large number of parameters with significant differences between the two groups.

In summary, from comparisons between survivors and non-survivors, this study indicated that the presence of predominant crazy-paving pattern on chest CT with the high and rapidly increased CT scores may help to identify the patients at high risk of developing ARDS before clinical deterioration. A larger, prospective study is required to confirm these findings with the more accurate quantitative assessment modality of the CT images in COVID-19.

### Ethical approval

This retrospective study was approved by the Ethics of Committees of Union Hospital, Tongji Medical College, Huazhong University of Science and Technology (No. 2020-0026), and followed the 1964 Helsinki Declaration and its later amendments or comparable ethical standards.

### Patient and other consents

Informed consent/deceased patient permission form for this retrospective study was waived by Ethics of Committees of Union Hospital, Tongji Medical College, Huazhong University of Science and Technology because only the anonymous data was collected and analyzed to facilitate better clinical decisions and treatment.

## Supplementary information

Supplementary file1

## Data Availability

The datasets generated in the current study are available from the corresponding author on request.

## Abbreviations

COVID-19: Coronavirus Disease 2019
CT: Computed tomography
SARS-CoV2: Severe acute respiratory syndrome coronavirus 2
RT-PCR: Real-time reverse transcription-polymerase chain reaction
ARDS: Acute respiratory distress syndrome
GGO: Ground-glass opacity
IQR: Inter-quartile range
