# Supplementary material for: Different computed tomography patterns of Coronavirus Disease 2019 (COVID-19) between survivors and non-survivors
Source: Sci Rep. 2020 Jul 9;10:11336. doi: 10.1038/s41598-020-68057-4 (PMC7347874; doi:10.1038/s41598-020-68057-4)
Supplement: Supplementary file 1 — Supplementary file1 [file 41598_2020_68057_MOESM1_ESM.pdf]

Full Title

Different computed tomography patterns of Coronavirus Disease 2019 (COVID-19) between survivors and non-survivors

Authors

Feng Pan, MD[*1,2], Chuansheng Zheng, Prof, MD, PhD[*1,2], Tianhe Ye, MD[1,2], Lingli Li, MD[1,2], Dehan Liu, MD[1,2], Lin Li, MD[1,2], Richard L. Hesketh, MD, PhD[3], Lian Yang, Prof, MD[1,2].

*These two authors contributed equally to the article.

[1]Department of Radiology, Union Hospital, Tongji Medical College, Huazhong University of Science and Technology, Jiefang Avenue #1277, Wuhan, 430022, China.

[2]Hubei Province Key Laboratory of Molecular Imaging, Wuhan, 430022, China.

[3]Department of Radiology, University College London Hospital, 235, Euston Road, London, NW1 2BU, UK.

Corresponding Author

Lian Yang, MD

Email: yanglian@hust.edu.cn

Supplementary Table S1. Curve estimations between surviror and non-survivor groups

	**Survivor group, n=83.**		**Non-survivor group, n=41.**	
**Equation**	**R^2**	**p value**	**R^2**	**p value**
Linear	0.247	<0.001	0.635	<0.001
Logarithmic[a]				
Inverse[b]				
Quadratic	0.455	<0.001	0.708	<0.001
**Cubic**	**0.545**	**<0.001**	**0.711**	**<0.001**
Compound[c]			0.659	<0.001
Power[a,c]				
S[b,c]				
Growth[c]			0.659	<0.001
Exponential[c]			0.659	<0.001
Logistic[c]			0.659	<0.001

Note: [a] If the independent variable (Time from the onset of initial symptoms (d)) contains non-positive values, the Logarithmic and Power models cannot be calculated; [c] if the independent variable (Time from the onset of initial symptoms (d)) contains values of zero, the Inverse and S models cannot be calculated; [d] If the dependent variable (total score) contains non-positive values, the Log transform cannot be applied and the Compound, Power, S, Growth, Exponential, and Logistic models cannot be calculated.
